# Peripheral Ulcerative Keratitis Associated with HCV-Related Cryoglobulinemia

**DOI:** 10.1155/2017/9461937

**Published:** 2017-07-12

**Authors:** Pedro Coelho, Carlos Menezes, Rita Gonçalves, Pedro Rodrigues, Elena Seara

**Affiliations:** ^1^Department of Ophthalmology, Hospital Pedro Hispano, Matosinhos, Portugal; ^2^Department of Ophthalmology, Hospital de Santa Luzia, Viana do Castelo, Portugal

## Abstract

**Purpose:**

To describe a case of peripheral ulcerative keratitis associated with type II cryoglobulinemia in the context of Hepatitis C infection.

**Methods:**

Case report and literature review.

**Results:**

A 36-year-old male patient presented to our emergency department with unilateral moderate pain in his right eye, associated with redness and photophobia. Medical background elicited a history of Hepatitis C and IV illicit drug abuse. Ocular examination revealed a BCVA of 20/30 of the affected eye and slight discomfort with eye movement. Biomicroscopy revealed a ring shaped peripheral corneal ulcer, with a dense white stromal infiltrate sparing the limbus, plus an adjacent area of mild anterior diffuse nonnecrotizing scleritis. No intraocular inflammation was present. Posterior segment was normal. The patient was placed under oral prednisolone (1 mg/Kg/day) with gradual tapering. A dramatic clinical response occurred, with complete resolution of the clinical condition. Systemic analytic workup aiming at autoimmune and infectious diseases was negative, except for high HCV-viral load and the presence of type 2 cryoglobulins.

**Conclusion:**

Albeit rare, cryoglobulinemia, even in the absence of systemic vasculitis, should be considered in the differential diagnosis of PUK, when systemic workout for autoimmune diseases is negative.

## 1. Introduction

Cornea and episclera are tissues with enhanced susceptibility to systemic inflammatory diseases, due to distinct local characteristics such as high vascular network of the conjunctiva and episclera, collagen riche-environment, and high number of Langerhans cells, immunoglobulin M (IgM), and complement C1, which favours immune complex formation [1]. As a consequence, in the context of PUK, it is mandatory to perform a systemic workout, since at least half of the cases are associated with a systemic disease, usually autoimmune connective tissue diseases (CTD) with a strong vasculitic component. Besides sight threatening, these disorders can be associated with increased mortality if left untreated, and PUK can occasionally be the first clinical manifestation. The pathology most frequently associated with PUK is rheumatoid arthritis (RA), accountable for more than 50% of the cases [2]. Other less frequent diseases include systemic lupus erythematosus, psoriatic arthritis, Wegener granulomatosis, polyarteritis nodosa, and Churg-Strauss syndrome, among others. PUK associated with systemic disease usually occurs in people older than 60 years and has a slight female predominance [2]. We report a case of PUK associated with type II cryoglobulinemia, an association sparsely reported in the literature [3].

## 2. Case Report

A 36-year-old male was brought to our emergency department with 5-day history of sharp pain, redness, and photophobia in his right eye (RE). There was no associated eye trauma or contact lens use. Ophthalmic charts were unremarkable until 8 years ago, in which are described four mild episodes of unilateral alternating PUK, more frequent in the winter. Medical background elicited a history of Hepatitis C for at least 10 years and a past of intravenous illicit drug abuse, namely, cocaine and heroin, discontinued when he was arrested and substituted by methadone (45 mg/day). There was no history of CDT.

**Figure 1:**
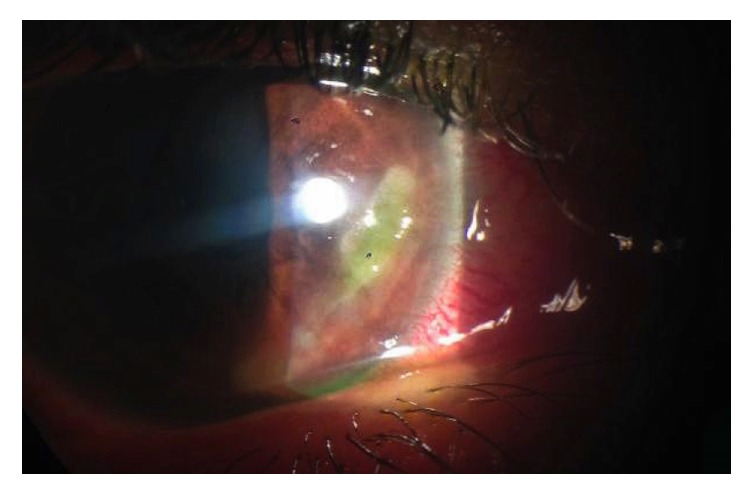
Corneal ulcer at presentation, ring shaped peripheral ulcer from 3 to 6 o'clock, sparing the limbus.

**Figure 2:**
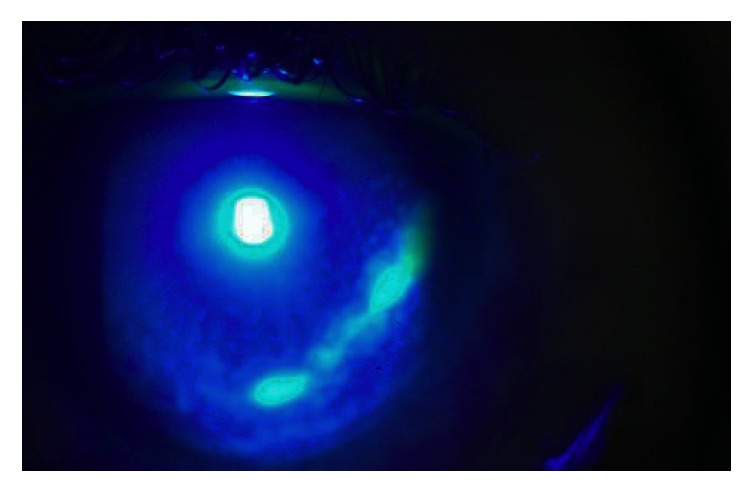
Fluorescein staining and cobalt blue filter, epithelial defect at presentation.

**Figure 3:**
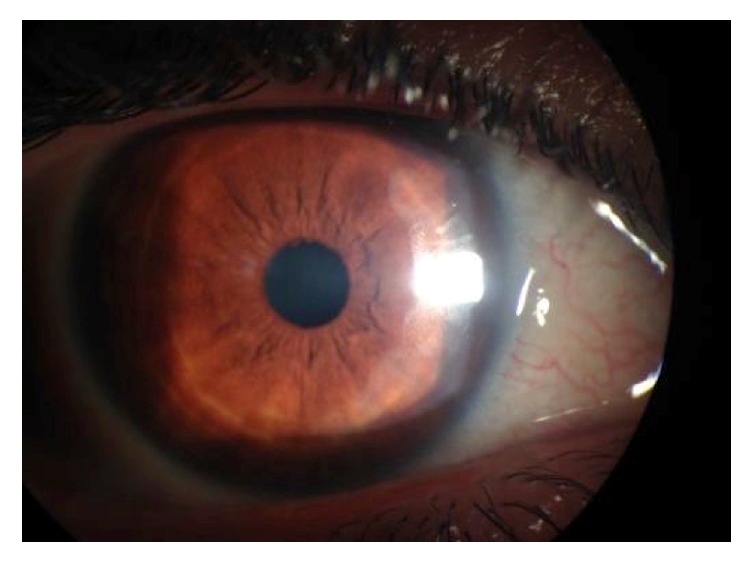
Two weeks after presentation, complete healing of corneal ulcer.

At the ophthalmological examination in the emergency department he presented a best-corrected visual acuity of 20/30 in the RE and 20/20 in his left eye (LE). Oculomotricity was normal, despite a mild pain in the RE. Biomicroscopy showed in the RE a deep conjunctival hyperemia, a ring shaped peripheral ulcer from 3 to 6 o'clock, with dense white stromal infiltrate and an adjacent area of mild diffuse nonnecrotizing scleritis (Figures 1 and 2). No signs of ocular rosacea or blepharitis were noted. Tyndall and flare were absent. IOP was 17 mmHg bilaterally. LE was unremarkable beside two discrete peripheral ring-like leucomas, sparing the limbus and without evident thinning. Posterior segment was normal in both eyes. After a 5-day course of topical ofloxacin 3 m/mL 2/2 hours + oxytetracycline ointment 5 mg/g 3x day, cyclopentolate 1% 2x day, and artificial tear, the patient was reassessed. Due to lack of clinical improvement systemic corticosteroids (CCT) were also started (oral prednisolone 1 mg/kg/day), with good clinical response. Follow-up evaluations evidenced a BCVA of 20/20, with complete epithelization, and resolution of anterior segment inflammatory signs in approximately two weeks (Figure 3). Systemic workout was negative for CTD (antinuclear antibodies, anti-double strand-DNA, ANCAs, Rheumatoid factor, Liver Kidney Microsomal Type 1 Antibodies, anti-SS-A/Ro, and SS-B/La), with normal complement levels. Additionally, infectious serology for syphilis, HIV, and Hepatitis B virus and Interferon-Gamma Release Assays (IGRA) was also negative. Blood cell count, urinalysis, and hepatic function were normal although with twofold increase in hepatic transaminases. HCV strain was genotype 1a with a RNA viral load over 2,6 million/mL. Cryoglobulin screen revealed mixed type 2 cryoglobulins without rheumatoid factor activity (86 *μ*g/mL), suggesting the diagnosis of PUK in the context of HCV-related cryoglobulinemia. No evidence of active vasculitis was present, namely, constitutional symptoms, purpure, arthralgia, membranous glomerulonephritis, and peripheral neuropathy. Patient was intended to start treatment with pegylated interferon plus ribavirin; however, lack of alcohol abstinence and sporadic illicit drug abuse have deferred treatment. After two years of follow-up, no recurrences of PUK or signs of small-vessel vasculitis were noticed.

## 3. Discussion

Cryoglobulins are immune complexes that precipitate at low temperatures and deposit in the endothelium, generating an inflammatory or occlusive response, and they can be found rarely in otherwise healthy people, at a very low concentration. If more than one immunoglobulin component is present, they are termed mixed cryoglobulins. Their prevalence, however, is far more common in HCV patient [4] and can lead to mixed cryoglobulinemia syndrome, a small to medium vessel vasculitis, resulting from production of polyclonal IgG and monoclonal (type II) or polyclonal (type III) IgM with rheumatoid factor activity [5]. This condition is associated with HCV infection in the large majority of patients, where a viral induced clonal B-cell expansion results in production of IgM that interacts with anti-HCV IgG and forms immune complexes [5].

The seasonality of the episodes, with winter predominance, the type II cryoglobulins in the serum of a patient with previous diagnosis of Hepatitis C, and the lack of CTD favour the diagnosis of PUK in the context of type II cryoglobulinemia. The absence of systemic signs of vasculitis does not exclude cryoglobulins as important element in PUK, as precipitation properties with local hyperviscosity are still present. A report by Johnson and Ohlstein, 2011 [3], describes a case of PUK and necrotizing scleritis induced by trauma in a patient with HCV-related cryoglobulinemia, without systemic involvement. It is known that the vast majority of patients with circulating cryoglobulins remain asymptomatic [4], although probably with higher sensitivity to trigger events. Also, PUK with associated scleritis may be the first clinical sign of MC [6].

Our patient experienced a dramatic improvement after starting systemic corticosteroids, despite the absence of systemic features associated with vasculitis, which further suggests the diagnosis of immunologically mediated PUK. This limited ocular involvement may explain the rapid response to corticosteroids monotherapy, similar to descriptions in previous reports [3]. Depending on the associated systemic disease, it is not infrequent the need to scale immunosuppressive therapy with cytotoxic (cyclophosphamide) or immunomodulating agents (methotrexate or ciclosporin), in order to halt the systemic and ocular inflammatory process.

Although there is scarce literature regarding ocular attainment in MC, it appears that anterior segment manifestations are the most common, with anterior scleritis and PUK being the most consistent findings. The physiological mechanism proposed to justify this anterior segment predominance is a temperature difference of 2°C cooler than the body normal temperature (<37°C), causing cryoglobulins to precipitate [7].

In conclusion, cryoglobulins should be measured in patients with HCV infection and signs of peripheral ulcerative keratitis even in the absence of vasculitis.
